# Case Report: Coexistent Wolff-Parkinson-White Syndrome and Brugada Phenocopy in a Patient With Pneumonia and Myocarditis

**DOI:** 10.3389/fcvm.2021.711364

**Published:** 2021-10-21

**Authors:** Li Wang, Yana Zhao, Lan Ma

**Affiliations:** ^1^Department of Electrocardiogram Diagnosis, Second Affiliated Hospital of Anhui Medical University, Hefei, China; ^2^Department of Cardiology, Sir Run Run Shaw Hospital, Zhejiang University School of Medicine, Hangzhou, China

**Keywords:** Wolff-Parkinson-White syndrome, Brugada phenocopy, fever, electrocardiogram (ECG), case report

## Abstract

**Background:** In recent years, Wolff-Parkinson-White (WPW) syndrome and Brugada electrocardiogram (ECG) patterns have been reported as coexistent in the same patient. In most cases, the two waveforms appeared separately. Here, we described combinations of different waveforms on one ECG, such as the Brugada pattern with delta waves and the Brugada pattern with paroxysmal supraventricular tachycardia (PSVT). Importantly, we recorded an alternate conversion of these combined ECG waveforms, which has not previously been reported in the literature. At the same time, we confirmed that the change in the waveform was related to fever by analyzing Holter data.

**Case:** A 48-year-old male was admitted to our hospital due to palpitations and fever. The patient had a history of a cold 3 days ago. Laboratory examinations showed an elevated neutrophil percentage (85%) and troponin I level (0.86 ng/ml). A chest computed tomography (CT) scan showed inflammation in the right lung. The diagnosis of pneumonia and myocarditis was made. ECG indicated WPW syndrome and the Brugada pattern. We recorded the dynamic changes in this combination of delta waves and Brugada waves with a Holter monitor, and we found the changes would happen when the patient's body temperature rose. The doctors thought that the patient's pulmonary infection led to fever, which caused the changes in waveform. After treatment with antibacterial therapy and supportive care, his body temperature returned to normal. The various laboratory indicators also gradually returned to normal. The doctor recommended that the patient undergo further pre-excitation bypass radiofrequency ablation treatment, but the patient refused and was discharged.

**Conclusion:** Delta waves and Brugada ECG patterns could appear on one ECG at the same time. There were dynamic changes of QRS complex, relating to fever.

## Introduction

The classic WPW syndrome includes PSVT and delta waves within sinus rhythm, which are blunt and located at the beginning of the QRS complex. Its presence indicates that there is an atrioventricular sideway, which is one of the anatomical conditions of PSVT, and the diagnosis of WPW syndrome can be established if there are indications of both a delta wave and PSVT in a patient's ECGs (1–3).

Brugada waves are a typical ECG appearance in patients with Brugada syndrome. There are a high take-off ST-segment elevation (≥2 mm) and inverted T wave in the leads V1 through V3, which is defined as type I Brugada pattern. Both are relatively rare arrhythmias. It is very rare for these two waveforms to appear on the same ECG.

Here, we report a case in which the patient's ECG showed the combinations of these two waveforms.

## Manuscript Formatting

### Case Description

A 48-year-old male was admitted to our hospital due to palpitations and fever. These symptoms were accompanied by fever but no chest pain, cough or expectoration. The patient had a history of a cold 3 days prior. He denied a personal history of heart disease and a family history of sudden death. After admission, the patient's temperature was 38.1°C, and his blood pressure was 148/87 mmHg. Rales were heard in the right lung. His heart rhythm was regular, and no pathological murmurs were heard in any valve.

### Diagnostic Assessment

The patient's first troponin I level was 0.86 ng/ml, and the neutrophil percentage was 85%. Echocardiography showed mild mitral regurgitation and left ventricular diastolic dysfunction, without segmental movement abnormalities and abnormal atrial and ventricular structures (see Supplementary Figures 1–12). The patient underwent three chest computed tomography (CT) scans taken 1, 4, and 12 days after admission. The CT examinations showed that the pneumonia in the upper right lung progressively worsened (Supplementary Materials).

**Figure 1:**
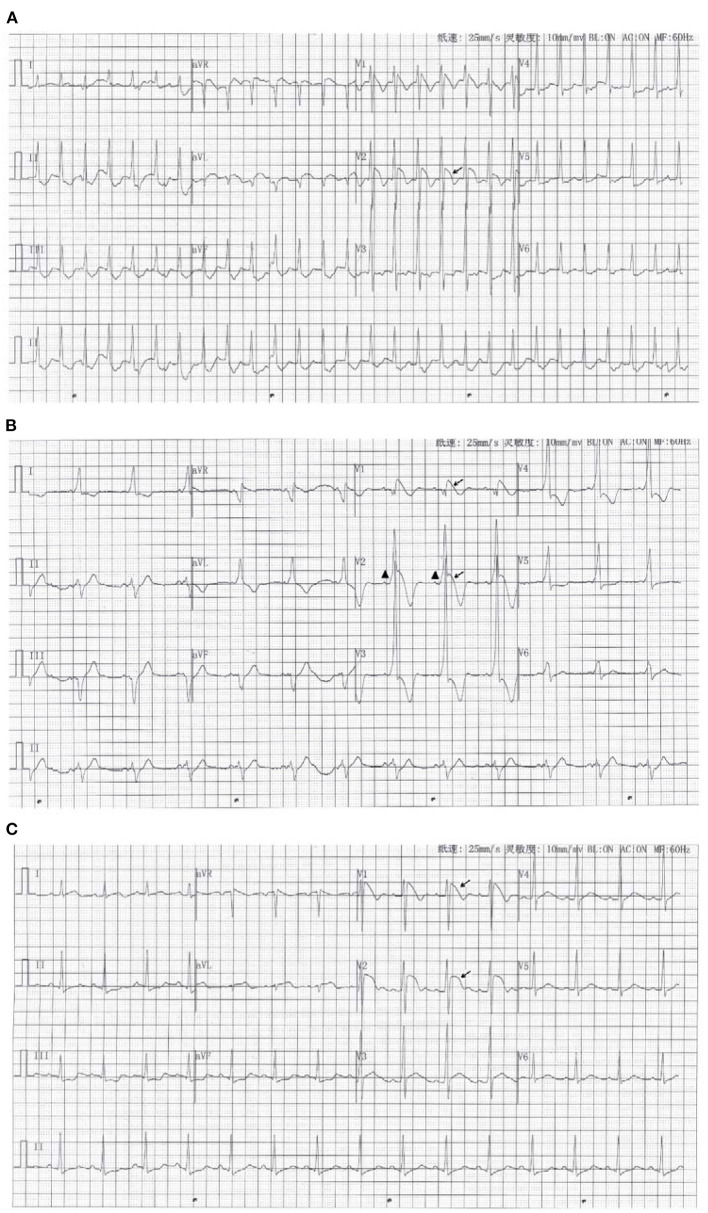
**(A)** The ECG recorded in the emergency room when the patient felt palpitations. It showed the coexistence of paroxysmal supraventricular tachycardia and the Brugada pattern (arrow) on one ECG. **(B)** The ECG with the delta wave (Triangle mark) and Brugada pattern (arrow). **(C)** The ECG with only the Brugada pattern (arrow).

**Figure 2:**
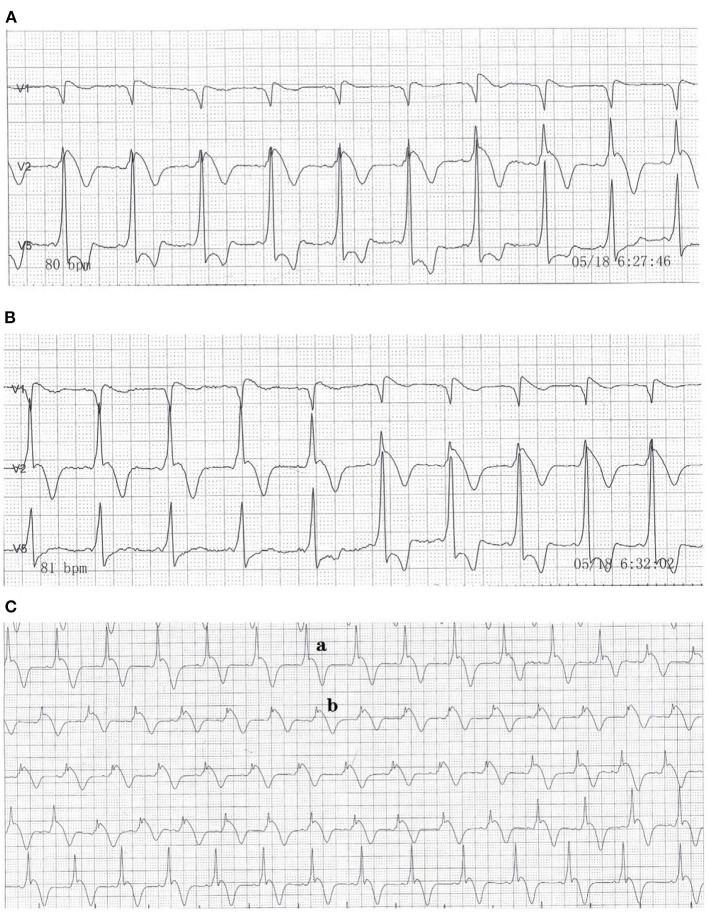
Dynamic electrocardiogram that shows one cycle of the QRS complex. **(A)** The QRS complex began to change in leads V1, V2, and V5 at 6:27 a.m. **(B)** The change ended at 6:32 a.m. **(C)** Dynamic electrocardiogram that shows the continuous changes in the QRS complex in lead V2.

The main challenges came in the form of the rare abnormality in his ECGs. There were Several ECGs and one 12-lead 24 h Holter monitor test were obtained. We identified the coexistence of paroxysmal supraventricular tachycardia (PSVT) and the Brugada wave or a delta wave and Brugada pattern in lead V2 (Figures 1A,B). Figure 1C shows a Brugada pattern without a delta wave. The height of the R wave was inversely proportional to the height of the ST-segment elevation (Figures 2A,B). The amplitude of the R wave had a cyclic course that moved from high to low and then back to high. The ST-segment showed the opposite changes (Figure 2C). These changes always happened during the night and in the early morning. Each cycle lasted from several seconds to several minutes. This phenomenon has not been reported thus far in the medical literature, and the cause is unclear.

Based on the patient's lung CT and elevated troponin, the diagnosis of pneumonia and myocarditis was made. The patient's ECGs recorded significant delta waves and PSVT, so the diagnosis of WPW syndrome was also confirmed. At the same time, the patient had no history of underlying heart disease and sudden death of family members. The typical Brugada pattern appeared on the ECG during fever, and it disappeared after the body temperature dropped. Therefore, we made the diagnosis of Brugada phenocopy. Clinicians thought the patient's pulmonary infection was the cause of the myocarditis. Fever caused by lung infection in turn caused the Brugada pattern.

### Therapeutic Intervention

**Figure 3:**
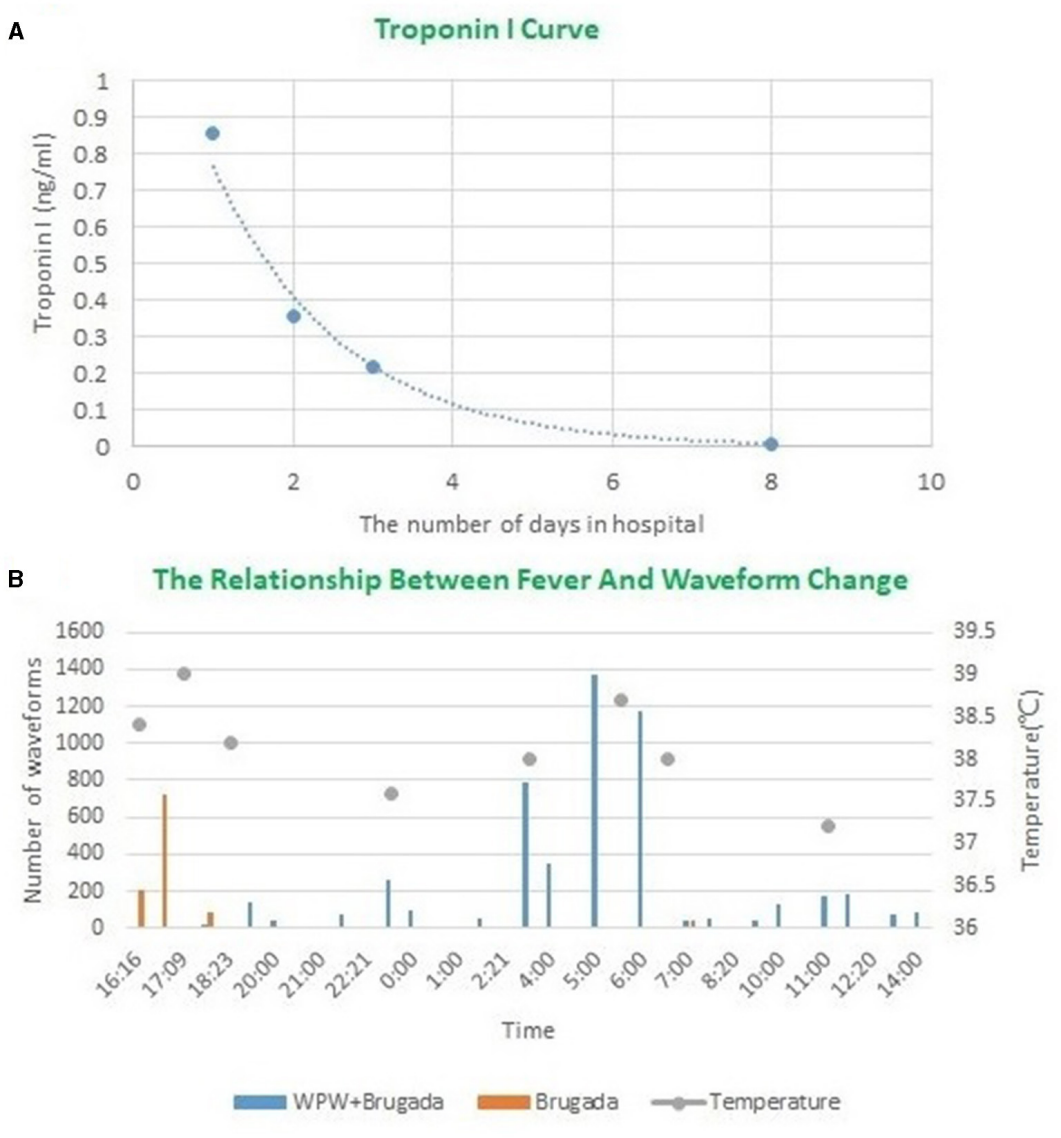
**(A)** The change curve of troponin I during hospitalization. **(B)** The relationship between fever and waveform changes during 24 h Hortor monitoring period on the second day of admission. The orange mark represents Figure 1C. The blue mark represents Figure 2Cb.

**Figure 4:**
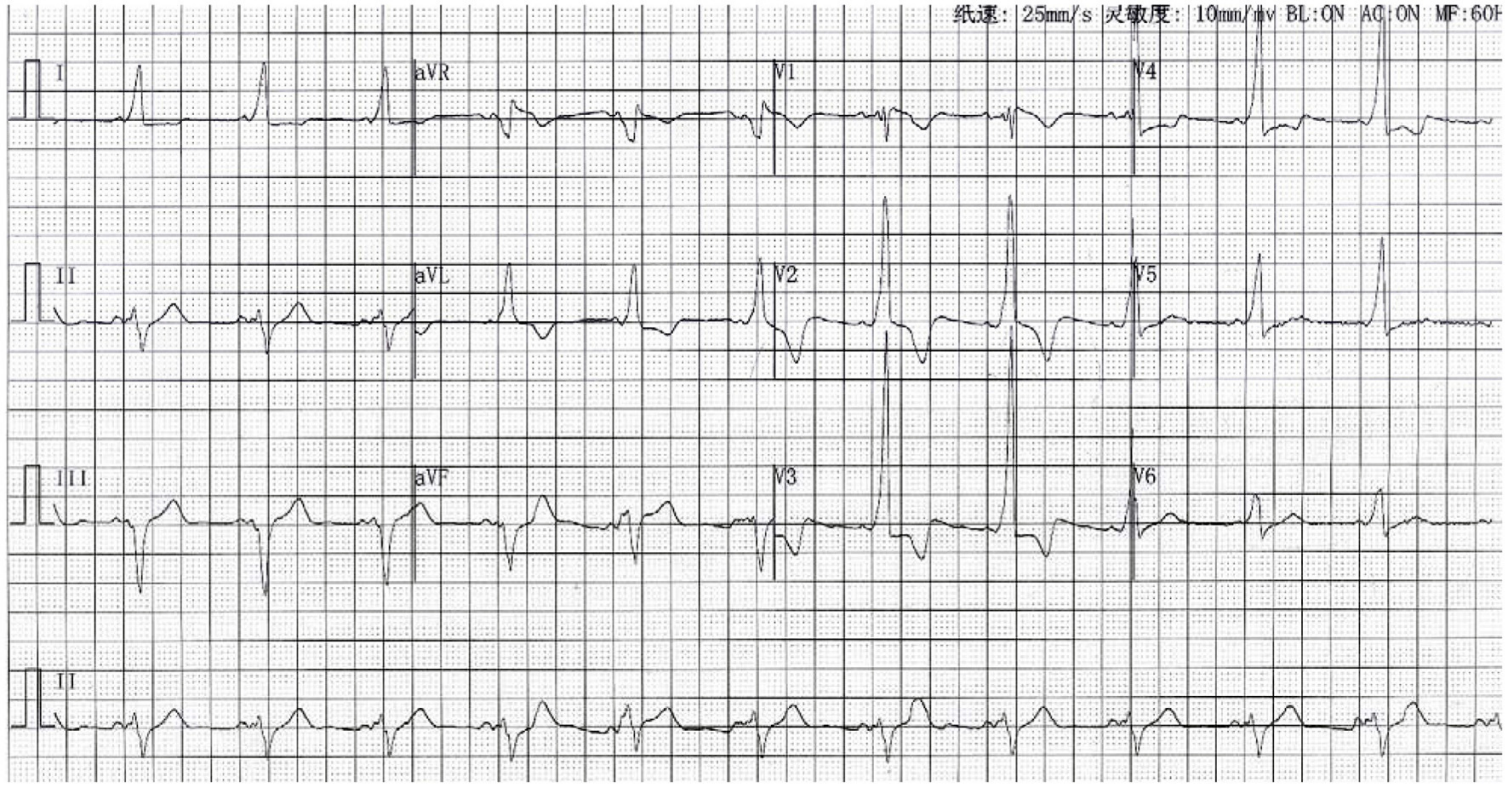
The 12-lead ECG after antibacterial treatment.

We were not sure whether this variability in the ECG could increase the patient's risk of sudden death. Therefore, the clinicians decided to treat the patient with antibiotics first instead of bypass ablation therapy. After the initiation of treatment, his body temperature and laboratory indicators gradually became normal (Figure 3A and Supplementary Materials). A subsequent ECG showed that the ST-segment in lead V2 had returned to baseline, while the delta wave remained (Figure 4). The patient's chest CT scan performed 12 days after admission showed that his lungs were still inflamed, indicating that he needed to continue anti-infective treatment; however, the patient refused and was discharged. Ten days after discharge from the hospital, the patient had a repeated chest CT. The chest CT scan showed that the inflammation in the upper right lung had disappeared (Supplementary Materials). One One year later, the patient was called to a follow up that not documented major aritmia except for occasional palpitations. We recommend that patients follow up another ECG to rule out the risk of sudden cardiac death, but he refused.

## Discussion

WPW syndrome is a clinical syndrome easily accompanied by tachyarrhythmia, also called pre-excitation syndrome. Impulses from the sinus node are transmitted down through additional channels to activate part of the ventricles earlier, causing pre-excitation in part of the ventricular muscles. This pre-excitement of the local myocardium manifests as a delta wave on the ECG, and PSVT is one of the tachyarrhythmia caused by pre-excitation syndrome. Therefore, WPW syndrome could be identified once delta wave and PSVT were recorded in one patient's ECGs.

Brugada phenocopy was first proposed by Baranchuk et al. (4), which is different from Brugada syndrome. Brugada syndrome is a hereditary ion channel disease that will causes arrhythmia. It has a higher risk of ventricular tachycardia and ventricular fibrillation. However, patients with Brugada phenocopy do not have congenital Brugada syndrome but present with the type I Brugada ECG pattern under certain specific conditions, such as exposure to some drugs (including IA and IC antiarrhythmic drugs), myocardial ischemia, fever, and other illnesses. When these triggers disappear, the ECG then returns to normal.

WPW syndrome with a concurrent Brugada wave is rarely reported. In a previous report, Eckardt et al. (5) observed 35 patients with Brugada syndrome, and they found that 10 patients (29%) had supraventricular tachyarrhythmia. Bodegas et al. (6) reported a case of right posterior septal pathway in a patient with Brugada syndrome. However, most of these had single delta waves or Brugada waves on the same ECG.

In our case report, a type 1 Brugada pattern and a ventricular pre-excitation phenomenon appeared concurrently on one ECG. In addition, we recorded the repeated cycles of these waves. In order to explore the relationship between fever and QRS waveform changes, we re-analyzed 24 h Holter monitor, found that the waveform changes always occurred when the body temperature rose. When the body temperature dropped below 37°C, the QRS complex was relatively stable, as shown in Figure 2Ca. When the body temperature exceeded 37°C, the QRS complex became more variable. We selected two of the most representative waveforms, Figures 1C, 2Cb, which meant that the QRS complex had changed once these two graphs appeared. Then we used dynamic analysis software to count the number of occurrences of the two waveforms per hour, and drew a combination diagram to reveal the relationship between the two waveforms distribution and body temperature (Figure 3B). From this figure, we could see that the period of waveform change and the period of fever was basically match. Therefore, fever was related to the waveform change. Comparing the waveforms before and after the change, the main difference was that the characteristics of Brugada pattern in the changed waveforms were more obvious. It also proved that fever was a powerful trigger of Brugada pattern (7). The dynamic variability in this association has not been reported thus far in the medical literature. And for the first time, we used statistical data to confirm the relationship between the waveform changes and fever.

The most important limitation of this study was the failure to perform intracardiac electrophysiological examinations to determine if the patient had Brugada syndrome. However, in combination with the patient's family history and his present history, the doctors strongly supported the diagnosis of “Brugada phenocopy.” Therefore, the correct analysis of the ECG and the comprehensive and accurate recording of the patient's medical history were particularly important.

Our case demonstrated that Coexistent WPW syndrome and Brugada phenocopy could appeared in one ECG, and appeared dynamic changes. These waveform changes was related to fever.

### Patient Perspective

In previous reports, the most common cause of Brugada phenocopy coexisting with WPW syndrome was the use of drugs (8). In our report, the reason was fever that was caused by bacterial infection. The patient's troponin I level was elevated, and there were many changes on his ECGs. There remains some uncertainty regarding whether this variability on ECG indicates can increase the risk of ventricular fibrillation or sudden death. These issues made us more conservative in our treatment decisions. The clinicians directed the patient to undergo radiofrequency ablation to eliminate the bypass after his condition became stable. However, the patient rejected the doctors' advice. In order to better evaluate the prognosis and risk, we included the patient in a follow-up plan.

## Data Availability Statement

The original contributions presented in the study are included in the article/Supplementary Material, further inquiries can be directed to the corresponding author/s.

## Ethics Statement

Written informed consent was obtained from the individual(s) for the publication of any potentially identifiable images or data included in this article.

## Author Contributions

LW analyzed and contributed to manuscript drafting. YZ and LM reviewed the literature and contributed to manuscript drafting. All authors have read and approved the final manuscript.

## Conflict of Interest

The authors declare that the research was conducted in the absence of any commercial or financial relationships that could be construed as a potential conflict of interest.

## Publisher's Note

All claims expressed in this article are solely those of the authors and do not necessarily represent those of their affiliated organizations, or those of the publisher, the editors and the reviewers. Any product that may be evaluated in this article, or claim that may be made by its manufacturer, is not guaranteed or endorsed by the publisher.
