# Supplementary material for: Case Report: Coexistent Wolff-Parkinson-White Syndrome and Brugada Phenocopy in a Patient With Pneumonia and Myocarditis
Source: Front Cardiovasc Med. 2021 Oct 21;8:711364. doi: 10.3389/fcvm.2021.711364 (PMC8566699; doi:10.3389/fcvm.2021.711364)
Supplement: Supplementary file 1 [file Data_Sheet_1.zip › Supplementary materials/Laboratory indicators/Changes in laboratory indicators.docx]

| Days*  Indicators | 1 | 2 | 3 | 4 | 5 | 6 | 7 | 8 |
| --- | --- | --- | --- | --- | --- | --- | --- | --- |
| Troponin I (ng/ml) | 0.86 | 0.36 | 0.22 |  |  |  |  | 0.01 |
| Glutamic-oxaloacetic transaminase (μ/l) | 83 |  |  |  | 72 |  |  | 76 |
| Creatine kinase (μ/l) | 2439 | 2929 | 1342 |  | 224 |  |  | 51 |
| Creatine kinase isoenzyme (μ/l) | 69 | 76 | 38 |  | 26 |  |  | 12 |
| Lactate dehydrogenase (μ/l) | 466 |  |  |  | 492 |  |  | 348 |
| White blood cell count (10^9^/l) | 7.35 |  |  |  | 3.63 |  | 4.82 |  |
| Percentage of neutrophils (%) | 85% |  |  |  | 58% |  | 54.8 |  |
| C reactive protein (mg/l) | 127.2 |  |  |  | 80.2 |  |  |  |
| Interleukin 6 (pg/ml) |  | 43 |  |  |  | 15 |  |  |
| Procalcitonin (ng/ml) |  | 0.299 |  |  |  | 0.134 |  |  |

*The number of days in hospital
