# Supplementary figures and images for: Case Report: Coexistent Wolff-Parkinson-White Syndrome and Brugada Phenocopy in a Patient With Pneumonia and Myocarditis
Source: Front Cardiovasc Med. 2021 Oct 21;8:711364. doi: 10.3389/fcvm.2021.711364 (PMC8566699; doi:10.3389/fcvm.2021.711364)

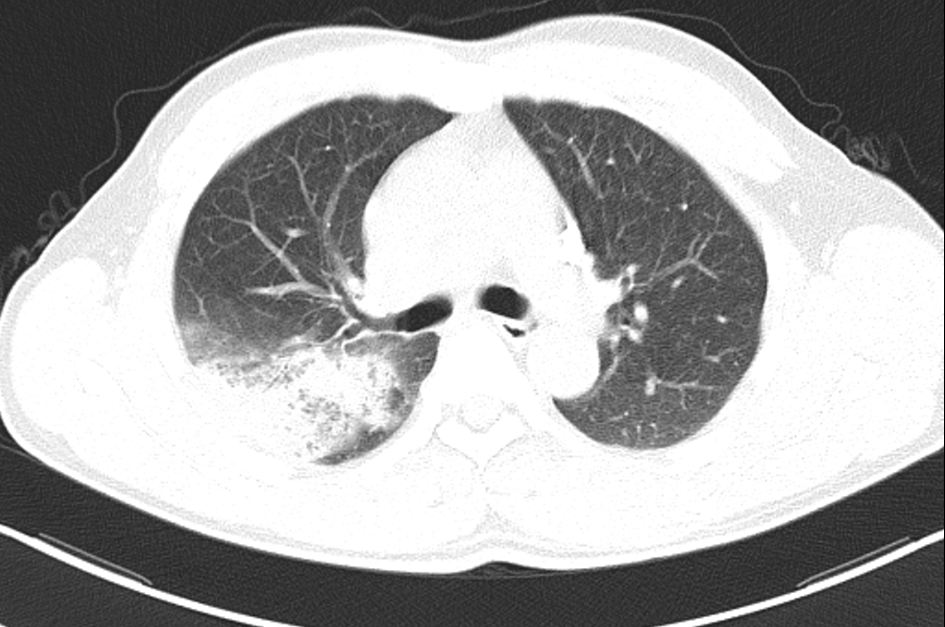

Supplement: Supplementary file 1 [file Data_Sheet_1.zip › Supplementary materials/CT/CT-A performed 1 day after admission.JPG]

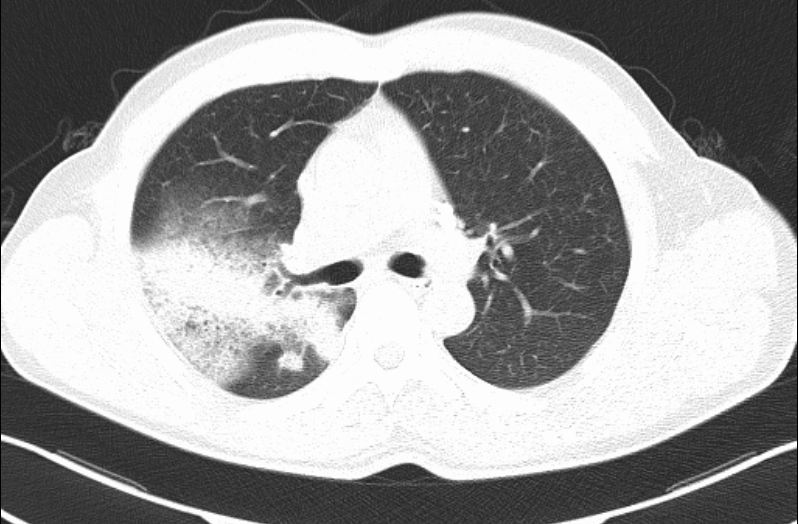

Supplement: Supplementary file 1 [file Data_Sheet_1.zip › Supplementary materials/CT/CT-B performed 4 days after admission.JPG]

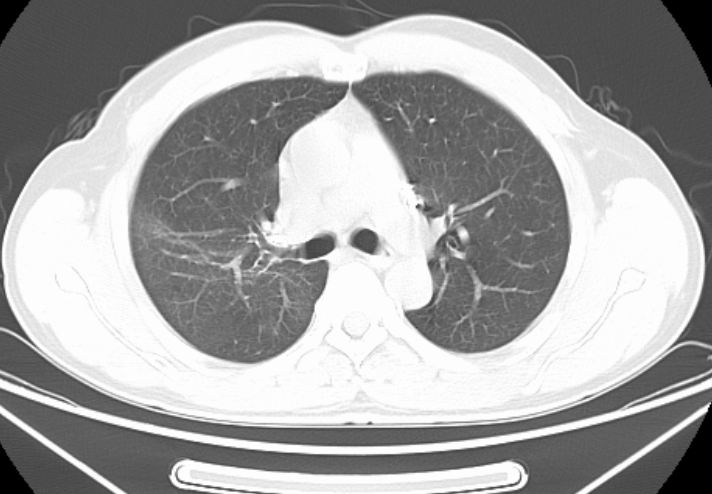

Supplement: Supplementary file 1 [file Data_Sheet_1.zip › Supplementary materials/CT/CT-D performed 10 days after discharge..JPG]

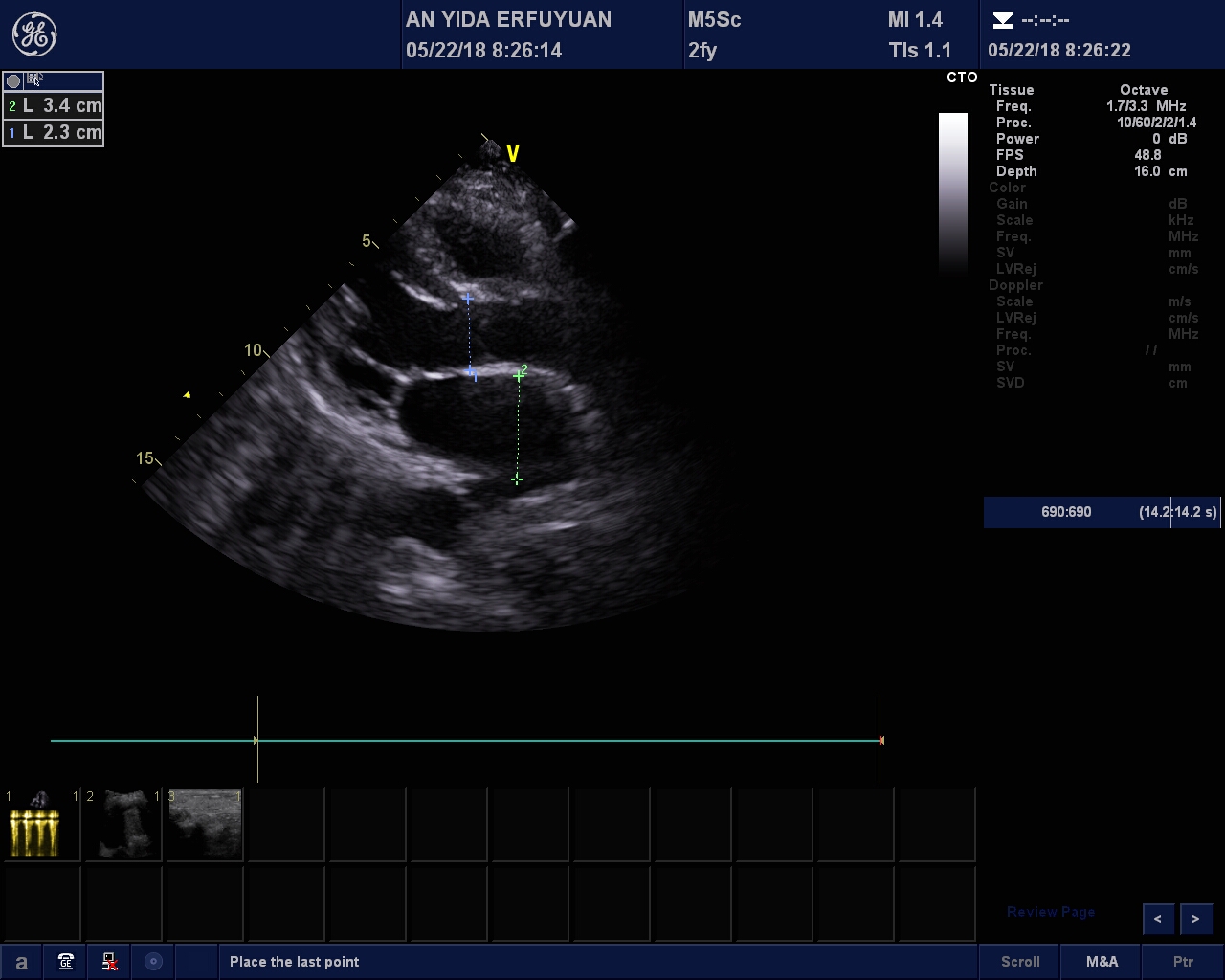

Supplement: Supplementary file 1 [file Data_Sheet_1.zip › Supplementary materials/Echocardiogram/Figure 1.jpg]

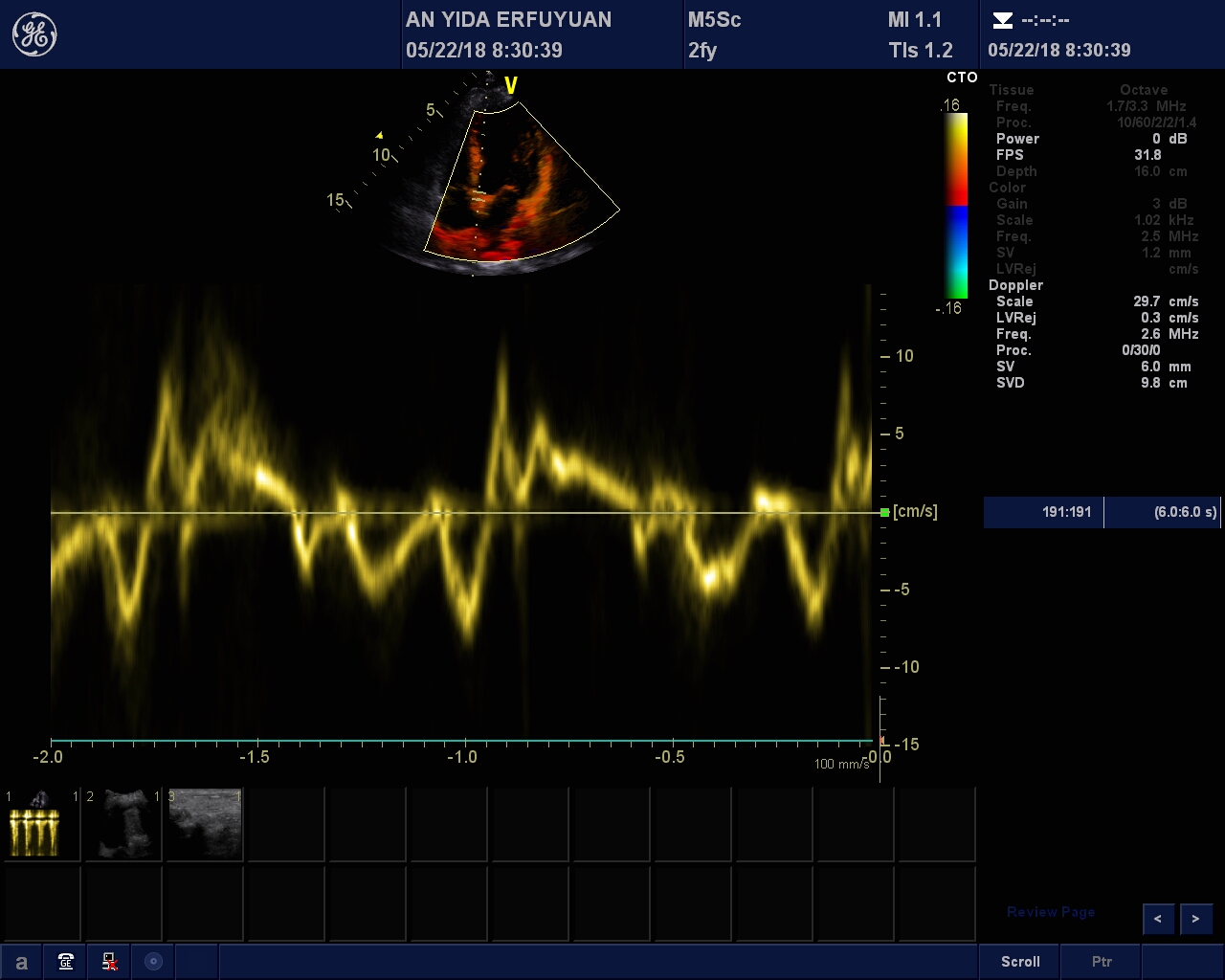

Supplement: Supplementary file 1 [file Data_Sheet_1.zip › Supplementary materials/Echocardiogram/Figure 10.jpg]

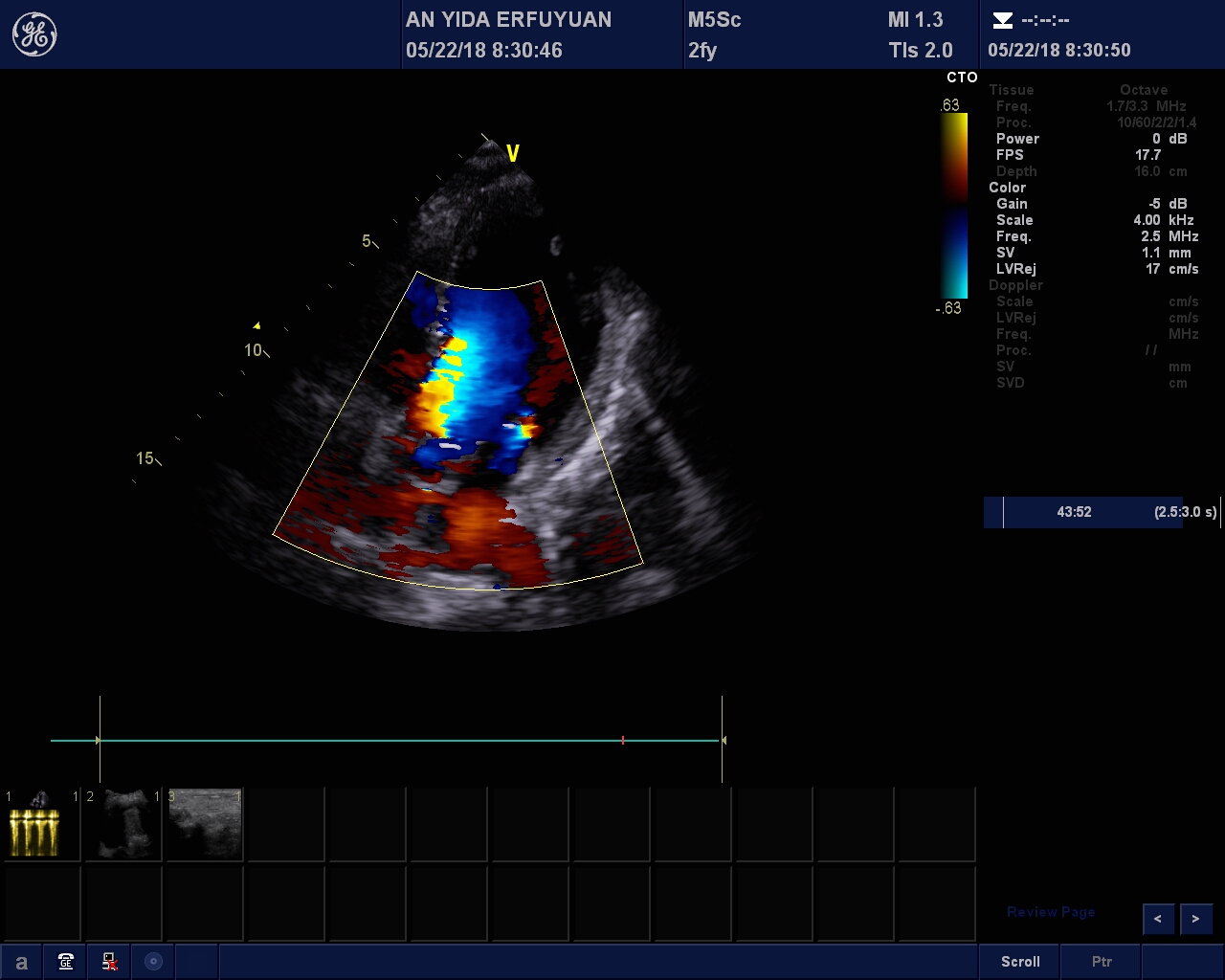

Supplement: Supplementary file 1 [file Data_Sheet_1.zip › Supplementary materials/Echocardiogram/Figure 11.jpg]

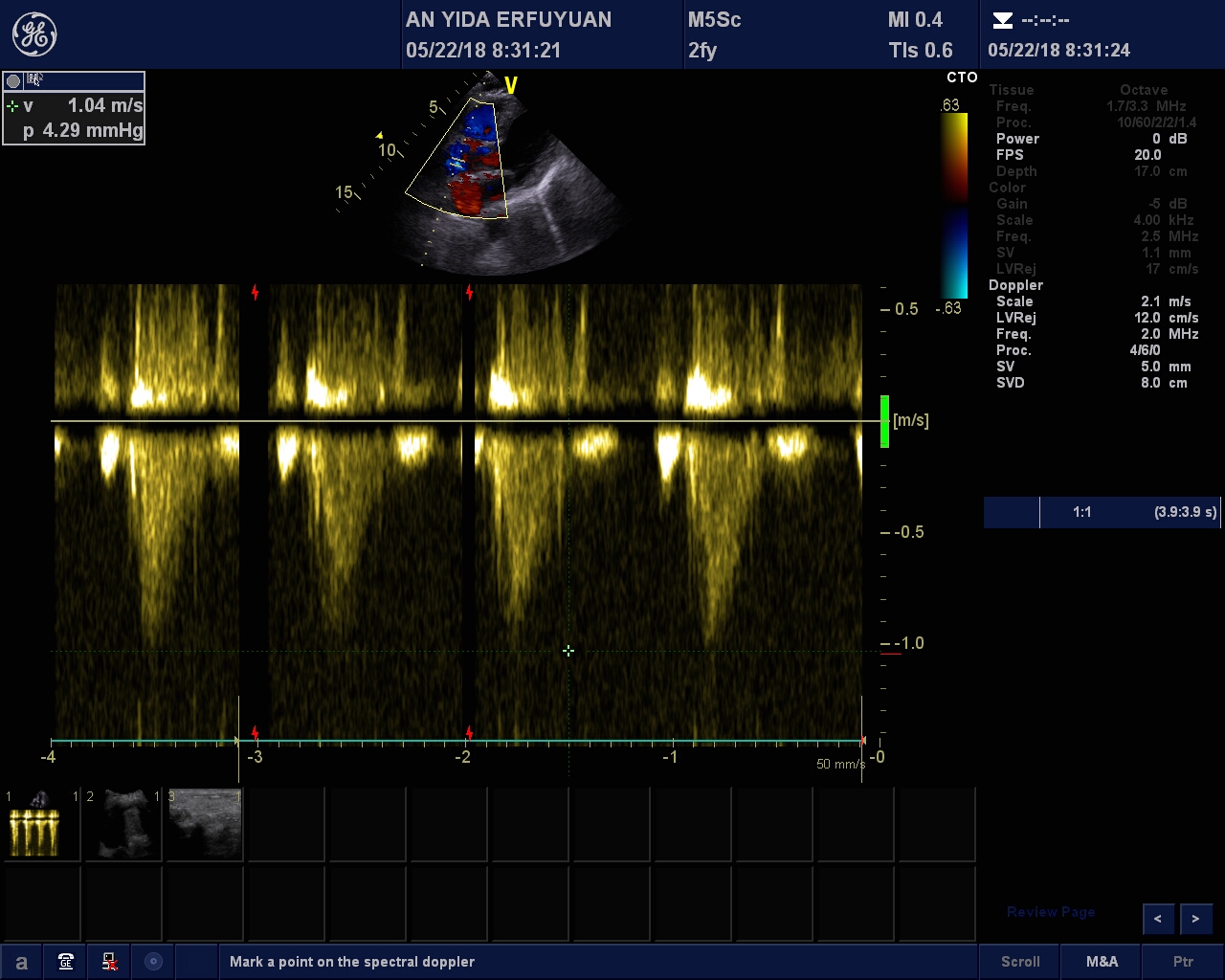

Supplement: Supplementary file 1 [file Data_Sheet_1.zip › Supplementary materials/Echocardiogram/Figure 12.jpg]

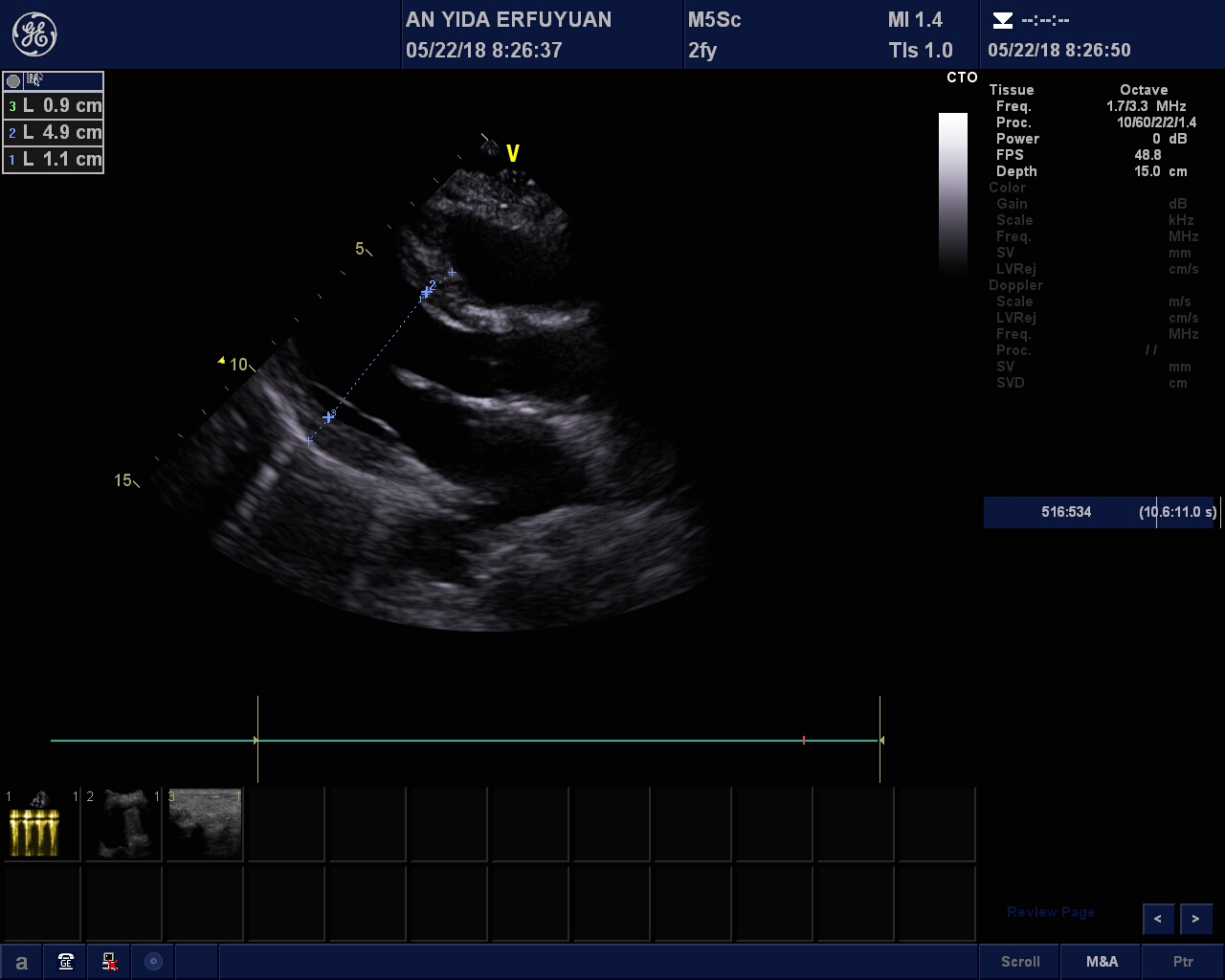

Supplement: Supplementary file 1 [file Data_Sheet_1.zip › Supplementary materials/Echocardiogram/Figure 2.jpg]

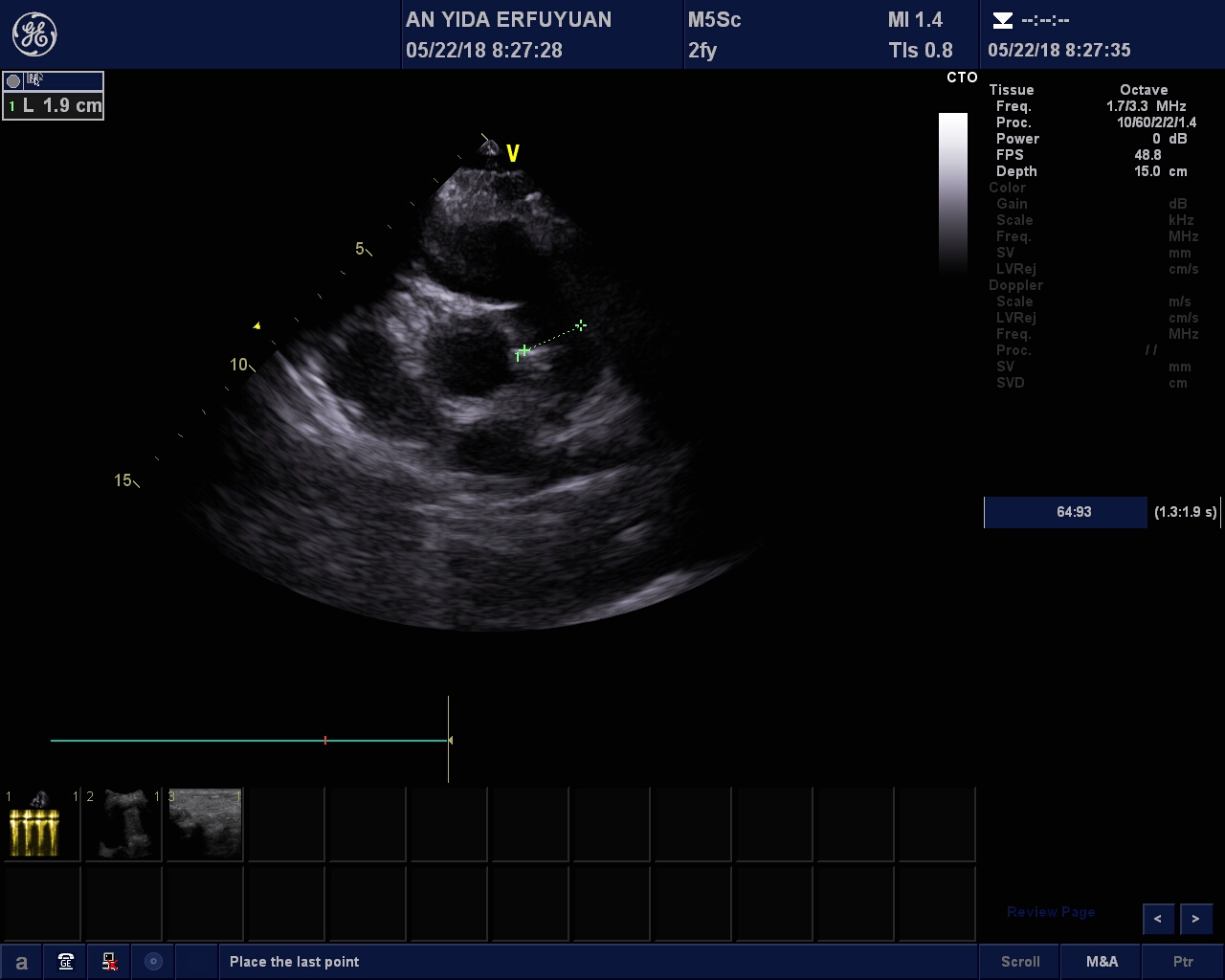

Supplement: Supplementary file 1 [file Data_Sheet_1.zip › Supplementary materials/Echocardiogram/Figure 3.jpg]

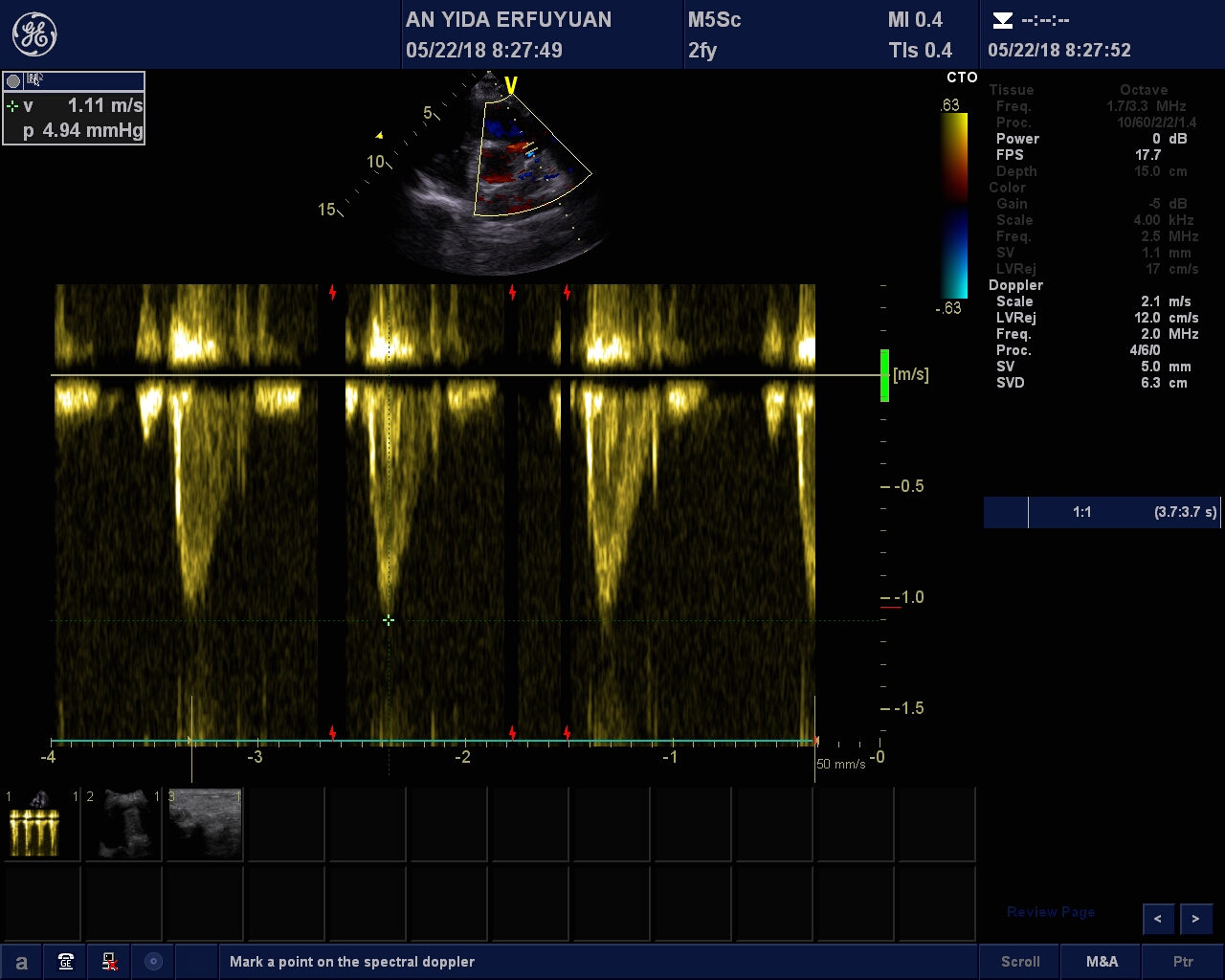

Supplement: Supplementary file 1 [file Data_Sheet_1.zip › Supplementary materials/Echocardiogram/Figure 4.jpg]

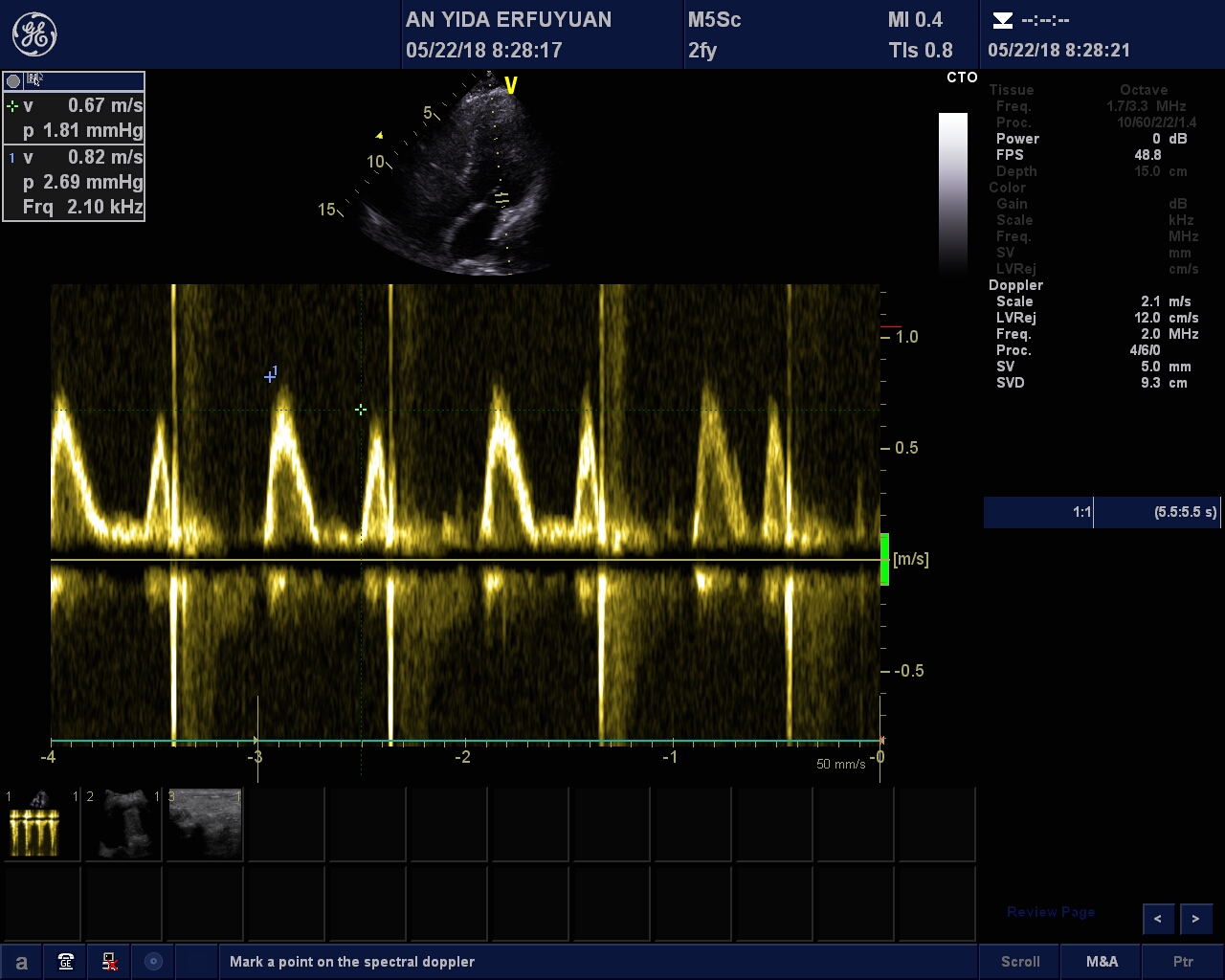

Supplement: Supplementary file 1 [file Data_Sheet_1.zip › Supplementary materials/Echocardiogram/Figure 5.jpg]

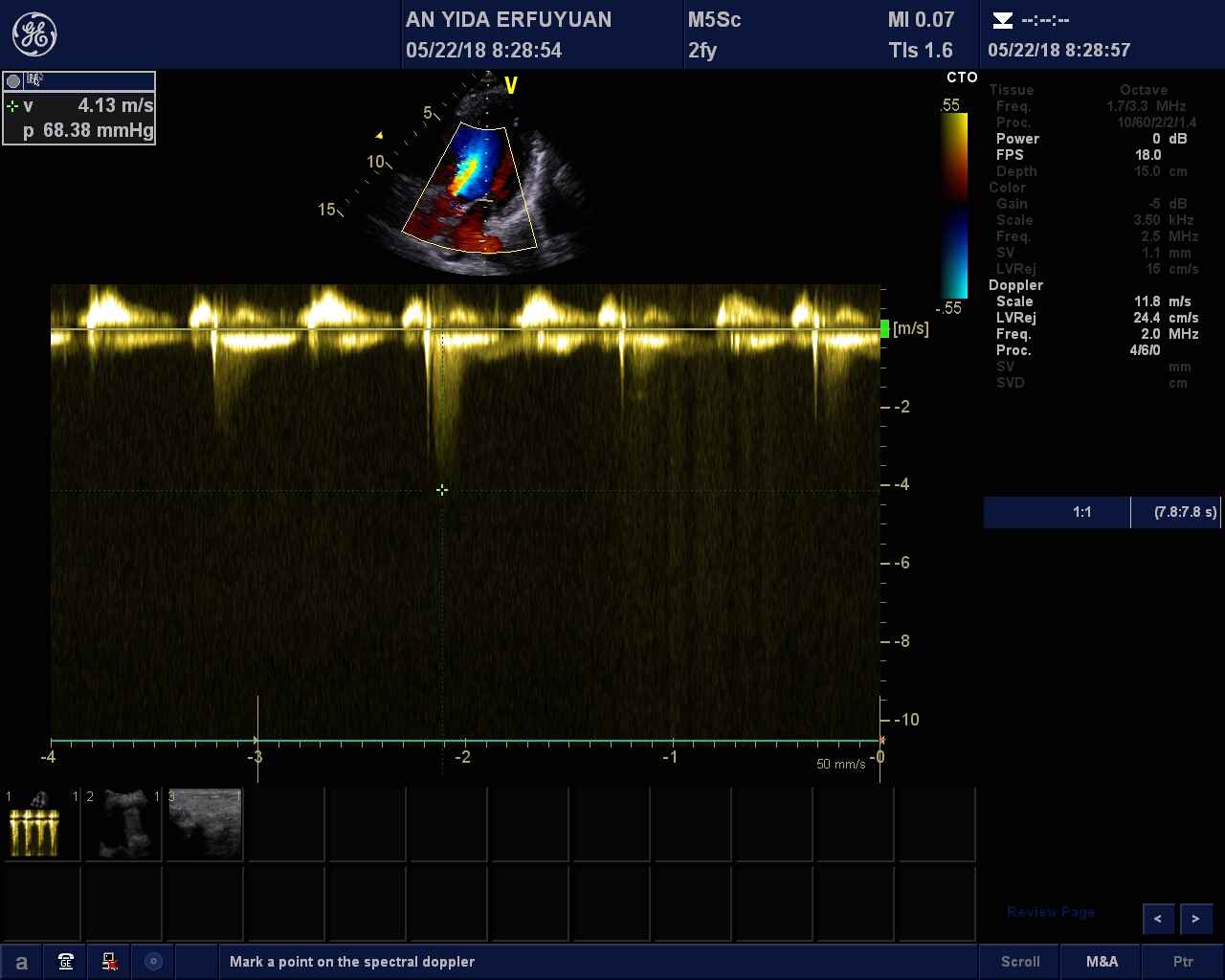

Supplement: Supplementary file 1 [file Data_Sheet_1.zip › Supplementary materials/Echocardiogram/Figure 6.jpg]

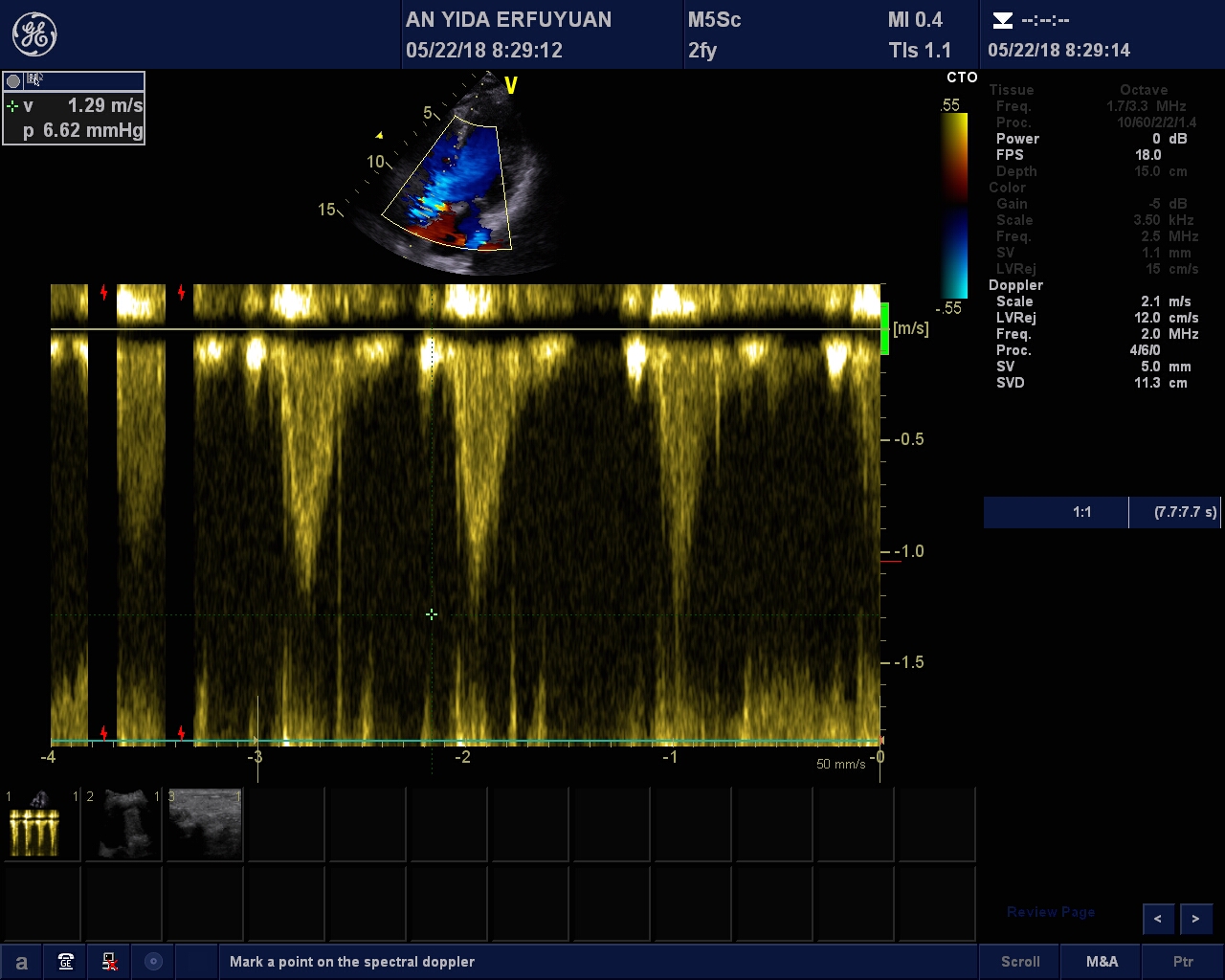

Supplement: Supplementary file 1 [file Data_Sheet_1.zip › Supplementary materials/Echocardiogram/Figure 7.jpg]

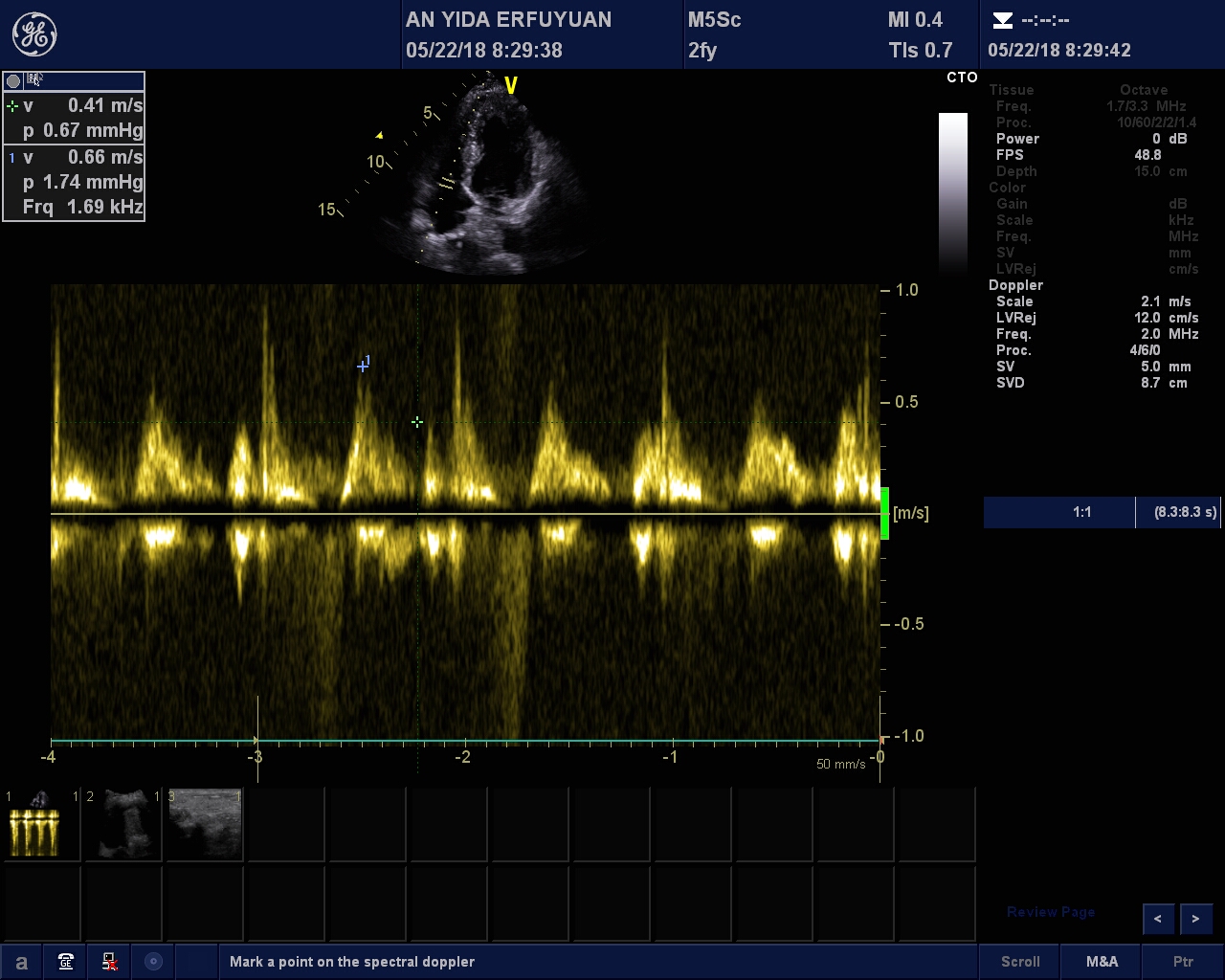

Supplement: Supplementary file 1 [file Data_Sheet_1.zip › Supplementary materials/Echocardiogram/Figure 8.jpg]

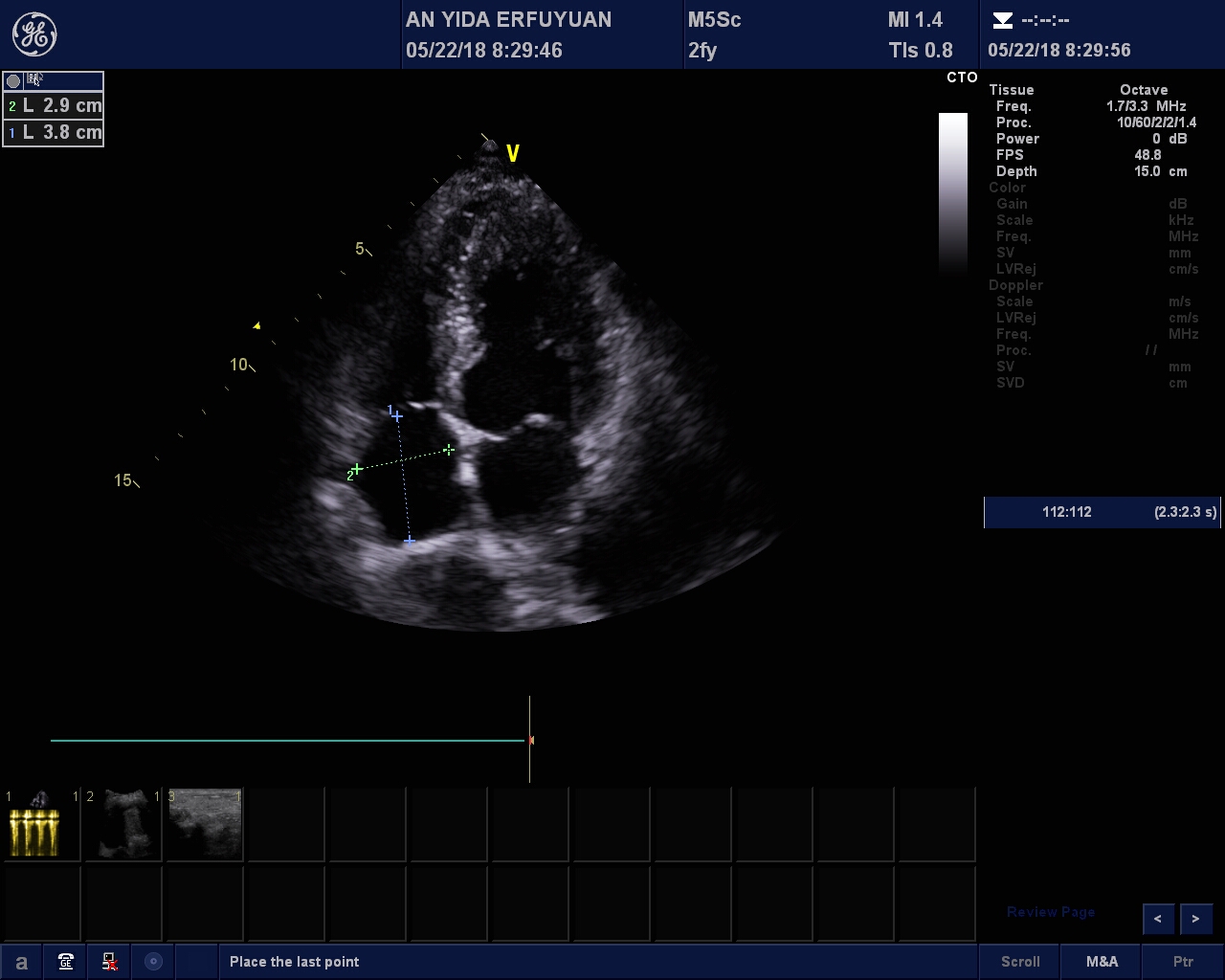

Supplement: Supplementary file 1 [file Data_Sheet_1.zip › Supplementary materials/Echocardiogram/Figure 9.jpg]
